# Economic costs, health-related quality of life outcomes and cost-utility of a physical and psychological group intervention targeted at older adults with neurogenic claudication

**DOI:** 10.1186/s12962-022-00410-y

**Published:** 2023-02-08

**Authors:** Maredza Mandy, Khan Kamran, Ioana R. Marian, Susan J. Dutton, Williamson Esther, Sarah E. Lamb, Petrou Stavros

**Affiliations:** 1 Division of Health Sciences, Warwick Medical School, The University of Warwick, Coventry, UK; 2 Warwick Clinical Trials Unit, University of Warwick, Warwick, UK; 3 Oxford Clinical Trials Research Unit, Nuffield Department of Orthopaedics, Rheumatology and Musculoskeletal Sciences, University of Oxford, Oxford, UK; 4 Exeter Medical School, University of Exeter, Devon, UK; 5 Nuffield Department of Primary Care Health Sciences, University of Oxford, Oxford, UK

**Keywords:** Economic costs, Health-related quality of life, Cost-effectiveness, Spinal stenosis, Neurogenic claudication, Rehabilitation, Exercise, Psychosocial

## Abstract

**Background:**

Emerging evidence suggests that structured and progressive exercise underpinned by a cognitive behavioural approach can improve functional outcomes in patients with neurogenic claudication (NC). However, evidence surrounding its economic benefits is lacking.

**Objectives:**

To estimate the economic costs, health-related quality of life outcomes and cost-effectiveness of a physical and psychological group intervention (BOOST programme) versus best practice advice (BPA) in older adults with NC.

**Methods:**

An economic evaluation was conducted based on data from a pragmatic, multicentre, superiority, randomised controlled trial. The base-case economic evaluation took the form of an intention-to-treat analysis conducted from a UK National Health Service (NHS) and personal social services (PSS) perspective and separately from a societal perspective. Costs (£ 2018–2019 prices) were collected prospectively over a 12 month follow-up period. A bivariate regression of costs and quality-adjusted life-years (QALYs), with multiple imputation of missing data, was conducted to estimate the incremental cost per QALY gained and the incremental net monetary benefit (INMB) of the BOOST programme in comparison to BPA. Sensitivity and pre-specified subgroup analyses explored uncertainty and heterogeneity in cost-effectiveness estimates.

**Results:**

Participants (N = 435) were randomised to the BOOST programme (n = 292) or BPA (n = 143). Mean (standard error [SE]) NHS and PSS costs over 12 months were £1,974 (£118) in the BOOST arm versus £1,827 (£169) in the BPA arm (*p* = 0.474). Mean (SE) QALY estimates were 0.620 (0.009) versus 0.599 (0.006), respectively (*p* = 0.093). The probability that the BOOST programme is cost-effective ranged between 67 and 83% (NHS and PSS perspective) and 79–89% (societal perspective) at cost-effectiveness thresholds between £15,000 and £30,000 per QALY gained. INMBs ranged between £145 and £464 at similar cost-effectiveness thresholds. The cost-effectiveness results remained robust to sensitivity analyses.

**Conclusions:**

The BOOST programme resulted in modest QALY gains over the 12 month follow-up period. Future studies with longer intervention and follow-up periods are needed to address uncertainty around the health-related quality of life impacts and cost-effectiveness of such programmes.

*Trial registration* This study has been registered in the International Standard Randomised Controlled Trial Number registry, reference number ISRCTN12698674. Registered on 10 November 2015.

**Supplementary Information:**

The online version contains supplementary material available at 10.1186/s12962-022-00410-y.

## Background

Treatment options for neurogenic claudication (NC) in older adults remain limited. Although medication and surgery are options, each possesses distinct disadvantages. Medication in older adults has potential side effects, including risk of falls, whilst surgery exposes older people to risk of wound infections and cardiorespiratory complications [1]. Physiotherapy is a viable alternative often offered as part of conservative care alongside medication [2]. However, the evidence base in support of physiotherapy is currently weak with published systematic reviews based on small, often single-centre trials, with short follow-up periods [2]. There is emerging evidence that interventions combining structured exercise and a cognitive behavioural approach can improve walking ability and physical function [3, 4]. However, data on the impact of these programmes on overall health-related quality of life (HRQoL) are limited and, to the best of our knowledge, the economic implications of delivering these types of structured programmes for older adults with NC have not been explored. Published evidence indicates that the costs of physiotherapy delivered, group-based, structured exercise programmes can be excessive based on data from other patient groups [5]. In the face of continued scarcity of health care resources, it is important to understand the economic costs, health benefits and cost-effectiveness of group-based exercise programmes in older patients with NC.

This study evaluates the economic outcomes, including the cost-effectiveness, of the Better Outcomes for Older people with Spinal Trouble (BOOST) programme, a physiotherapist delivered physical and psychological intervention for older adults with NC, compared to best practice advice (BPA).

## Methods

### Trial background

The BOOST Trial was a pragmatic, multicentre, superiority randomised controlled trial (RCT), and the protocol has been published previously [6]. In brief, community-dwelling adults, aged 65 years and over, who reported symptoms consistent with NC were eligible and were identified through spinal clinics (primary and secondary care) and general practice records. The trial had a pre-specified target sample size of 402 participants [6]. Recruitment occurred between 01 August 2016 and 29 August 2018 at 15 trial sites in England. Participants were randomised (2:1 ratio) to either the BOOST programme or BPA using a secure, telephone randomisation service. The primary clinical outcome was the Oswestry Disability Index (ODI) at 12 months and other important outcomes included pain, HRQoL outcomes, physical activity and strength [6]. Economic data were collected at baseline and as part of the study follow-up at 6 and 12 months.

### Comparator interventions

The experimental intervention was a combined physical and psychological group programme (BOOST programme) that included an individual assessment followed by a supervised component delivered by a physiotherapist in up to twelve 90 min group sessions over 12 weeks. Group sessions involved education and discussion using a cognitive behavioural approach (30 min) followed by individually tailored group exercises (60 min) [7]. The exercises targeted muscle strength, balance, and flexibility whilst the walking circuit aimed to increase walking self-efficacy, dynamic balance and mobility. Participants were introduced to twice-weekly home exercises during session five and asked to undertake these during and beyond the formal programme. Physiotherapists conducted follow-up telephone reviews approximately 1 and 2 months after completing the supervised sessions, to promote adherence with the home exercises.

The control intervention was BPA delivered during individual physiotherapy appointments. Each participant underwent an assessment of symptom presentation and walking ability to tailor the advice and education provided. Verbal and written advice and education were provided, including education about NC, being physically active, use of medications, when to seek more advice and prescription of up to four home exercises. The control intervention was ideally delivered in one session. However, if needed, two review appointments were permitted to re-enforce advice and review exercises or walking aids. Physiotherapists could not provide treatments such as manual therapy, acupuncture or structured exercise sessions.

### Overview of economic analyses

The economic analyses involved evaluation of economic costs, HRQoL outcomes and cost-effectiveness of the BOOST programme where cost-effectiveness was expressed in terms of incremental cost per quality-adjusted life year (QALY) gained. The base-case economic evaluation took the form of an intention-to-treat, imputed analysis conducted from a UK National Health Service (NHS) and personal social services (PSS) perspective in line with the National Institute for Health and Care Excellence (NICE) reference case [8]. The NHS payer perspective considers intervention-related treatment costs and other health service resource use and costs associated with the managing the disease whilst a personal social services perspective includes services provided by local authorities for several vulnerable groups, including older people. A 12 month time horizon for the economic evaluation was used mirroring the trial follow-up period and therefore no discounting was required.

### Costs

Three broad resource use and costs categories were estimated: (i) costs associated with each intervention delivery; (ii) health and personal social service use during the 12 months’ follow-up; and (iii) societal resource use and costs including economic values of lost productivity (e.g., lost income by participants and their carers) (Additional file 1: Table S1) (Appendix). All costs were expressed in pounds sterling and valued in 2018–19 prices. Where required, costs were inflated or deflated to 2018–19 prices using the NHS Cost Inflation Index (NHSCII) [9].

#### Intervention costs

Direct intervention costs were costs associated with the delivery of the BOOST programme. These included: (1) development and training costs; (2) staff costs including those associated with direct participant contact and non-contact time (i.e., time used to set up the sessions, indirect administrative activities, and intervention-related supervision activities); and (3) equipment costs (Additional file 1: Table S2) (Appendix). Unit costs for staff were obtained from the Personal Social Services Research Unit (PSSRU) Unit Costs of Health and Social Care 2019 compendium and were multiplied by the time taken to perform a specified activity (e.g., conduct a group session). All resource use data related to the group sessions were recorded on activity logs completed by physiotherapists and exercise assistants including: (i) the time taken to deliver sessions, (ii) number of participants in attendance and, (iii) number and grade of physiotherapists in attendance. Costs of equipment were obtained directly from the trial’s expenditure records and from the NHS Supply Chain Catalogue 2018 [10]. An annual equivalent cost of equipment was obtained by annuitising capital costs of each item over its useful life span, applying a discount rate of 3.5% per annum.

*NHS and PSS costs*. Participants (or their next-of-kin) reported health and social service resource use through questionnaires administered at 6- and 12 months post-randomisation. These resource inputs were valued using unit costs identified through national cost compendia in accordance with NICE’s Guide to the Methods of Technology Appraisal 2013 [8]. Unit cost data were derived based on the Department of Health and Social Care’s Reference Costs 2017–18 schedules [11], the PSSRU Unit Costs of Health and Social Care 2019 compendium [12], 2018 volumes of the British National Formulary [13], and the NHS Supply Chain Catalogue 2018 [10].

#### Societal costs

Analyses from a societal perspective additionally encompassed economic values for work absences (by patients and their caregivers), travel costs and privately incurred health expenditures. We included economic values of work absences by caregivers as caregivers of elderly frail people are potentially at increased risk of disrupted engagement within the labour market. Although data on the value of carers’ time is sparse, available data suggest that a 1% increase in hours of care translates, on average, into slightly more than a 1% decrease in hours of work [14]. Economic values of work absences were estimated as a product of the number of participant-reported days off work (for themselves and their caregivers) and national average daily earnings delineated by age, gender and occupational sector derived from the Office for National Statistics’ Annual Survey for Hours and Earnings [15]. Travel costs and privately incurred health expenditures were self-reported by trial participants.

#### Health-related quality of life outcomes

HRQoL was assessed using the EuroQol EQ-5D-5L instrument [16] completed at baseline, and at 6 and 12 months post-randomisation. The EQ-5D-5L instrument defines HRQoL in terms of five dimensions (mobility, self-care, usual activities, pain/discomfort, anxiety/depression), each with five levels of severity. Responses to the EQ-5D-5L descriptive system were mapped onto the EQ-5D-3L value set using the van Hout et al. interim cross-walk algorithm [17], as recommended by NICE in England and Wales [18]. Patient-level QALYs were estimated using the area under the curve approach, assuming linear interpolation between the utility scores, i.e., the preference-based values attached to the health states generated from the EQ-5D-5L descriptive system.

#### Handling of missing data

Multiple imputation by chained equations was used to predict missing costs and health utility scores based on the assumption that data were missing at random (MAR). The MAR assumption was tested through a series of logistic regression analyses comparing participants’ characteristics for those with and without missing endpoint data. Imputation was achieved using predictive mean matching, which has the advantage of preserving non-linear relationships and correlations between variables within the data. Fifty imputed datasets were generated to inform the base-case and subsequent sensitivity and subgroup analyses. Parameter estimates were pooled across the imputed datasets using Rubin’s rules [19] to account for between- and within-imputation components of variance terms associated with parameter estimates.

#### Cost-effectiveness analysis

Mean resource use, cost and health utility values were compared between the trial groups using two sample *t-*tests. Differences between groups, along with confidence intervals (CIs), were estimated using non-parametric bootstrap estimates (10,000 replications). Mean incremental costs and mean incremental QALYs were estimated using seemingly unrelated regression (SUR) methods that account for the correlation between costs and outcomes [20]. The SUR was adjusted for covariates (baseline utilities, gender). Following imputation, non-parametric bootstrap methods were used to generate the joint distribution of costs and outcomes and to populate a cost-effectiveness plane. The incremental cost-effectiveness ratio (ICER) for the BOOST programme was compared with BPA by dividing the between-group difference in adjusted mean total costs by the between-group difference in adjusted mean QALYs. Mean ICER values were compared against cost-effectiveness threshold values (i.e. society’s willingness to pay for an additional QALY) ranging between £15,000 and £30,000 per QALY gained in line with NICE guidance [21]. ICER values lower than the threshold are considered cost-effective for use in the UK NHS. The incremental net monetary benefit (INMB) of switching from BPA to the BOOST programme was calculated at each of the cost-effectiveness threshold values. The net monetary benefit is the economic benefit of an intervention (expressed in monetary terms) net of all costs. A positive incremental NMB suggests that, on average, the BOOST programme is cost-effective compared with BPA, at the given cost-effectiveness threshold.

### Sensitivity and subgroup analyses

Pre-specified sensitivity analyses were undertaken to assess the impact of alternative aspects of the cost-effectiveness of the BOOST programme and included restricting the analyses to complete cases (i.e. the sample of participants with no missing costs or outcome data at any time point) and replicating the analysis from a societal perspective. Pre-specified subgroup analyses were conducted by age (65–74 years/75 years +); gender (male/female); baseline ODI scores (< = 22, > 22); baseline Tilburg Frailty Index (TFI) scores (0–4, not frail/5 + , frail) (17); baseline Fear Avoidance Beliefs Questionnaire (FAB) scores (0–14, less fear/15 + , more fear) (18); and baseline hand grip strength (HGS) (men: < 30/30 + ; women < 20/20 +).

## Results

### Study population and data completeness

Baseline characteristics of participants were well-matched between the randomised groups (Table 1). The exception was the proportion of people classified as frail according to the TFI (11% higher in BPA group), but other markers of frailty including walking capacity (measured by the 6 Min Walk Test), physical performance (measured by the Short Physical Performance Battery) and HGS were similar. Complete QALY profiles were available for 357 (82%) participants based on the participant-reported EQ-5D-5L. Completion of health resource use data for the economic evaluation was similar at each time-point between the BOOST and BPA groups.

**Table 1:** Baseline characteristics (Mean (standard deviation) unless stated)

	BPA (n = 143)	BOOST programme (n = 292)	Overall (n = 435)
Descriptors			
Age (years) at baseline	75.0 (5.6)	74.8 (6.2)	74.9 (6.0)
Male, n (%)	60 (42.0%)	129 (44.2%)	189 (43.4%)
Female, n (%)	83 (58.0%)	163 (55.8%)	246 (56.6%)
Care requirements, n (%)			
Unpaid carer			
Yes	31 (21.7%)	54 (18.5%)	85 (19.5%)
No	112 (78.3%)	238 (81.5%)	350 (80.5%)
If yes, does unpaid carer live in?			
Yes	25 (17.5%)	41 (14.0%)	66 (15.2%)
No	6 (4.2%)	13 (4.5%)	19 (4.4%)
Paid carer			
Yes	6 (4.2%)	10 (3.4%)	16 (3.7%)
No	136 (95.1%)	280 (95.9%)	416 (95.6%)
Missing	1 (0.6%)	2 (0.6%)	3 (0.6%)
If yes, does paid carer live in?			
Yes	2 (1.4%)	4 (1.4%)	6 (1.4%)
No	4 (2.8%)	6 (2.1%)	10 (2.3%)
Employment status, n (%)			
Retired	125 (87.4%)	263 (90.1%)	388 (89.2%)
Semi-retired	6 (4.2%)	19 (6.5%)	25 (5.7%)
Employed	1 (0.7%)	3 (1.0%)	4 (0.9%)
Self-employed	3 (2.1%)	2 (0.7%)	5 (1.1%)
Unemployed	0 (0.0%)	0 (0.0%)	0 (0.0%)
Permanently sick or disabled	1 (0.7%)	2 (0.7%)	3 (0.7%)
Looking after home or family	2 (1.4%)	3 (1.0%)	5 (1.1%)
Other	5 (3.5%)	0 (0.0%)	5 (1.1%)
Health-related quality of life			
EQ-5D-5L utility score	0.580 (0.197)	0.603 (0.193)	0.596 (0.194)
Pain and disability			
Oswestry disability index	32.3 (14.2)	33.2 (13.7)	32.9 (13.9)
Frailty			
Tilburg frailty index	4.9 (2.5)	4.4 (2.7)	4.5 (2.6)
Classified as frail, n (%)			
Not frail	63 (44.1%)	156 (53.4%)	219 (50.3%)
Frail	80 (55.9%)	130 (44.5%)	210 (48.3%)
Missing	0 (0%)	6 (2.1%)	6 (1.5%)
Attitudes and beliefs			
Fear avoidance beliefs	12.7 (5.4)	13.0 (6.1)	12.9 (5.9)
Clinical assessment			
Six minute walk test	260.4 (101.3)	252.9 (98.1)	255.4 (99.1)
Hand grip strength (kg)	26.68 (10.53)	26.66 (10.47)	26.67 (10.47)
Short performance physical battery, median (IQR)	9 (8, 11)	9 (7, 11)	9 (8, 11)

With the exception of hospital outpatient services, data completion for categories of health resource use ranged between 75 and 88% (Table 2)

*IQR* Interquartile range

**Table 2:** Summary of data completeness of economic measures

Health economic variable by time point	Treatment arm			
	Group physiotherapy programme (N = 292)		Best practice advice (N = 143)	
	Completed^i^ (n)	Completed (%)	Completed (n)	Completed (%)
Hospital inpatient services				
6 months post-randomisation	243	83.22%	115	80.42%
12 months post-randomisation	221	75.68%	115	80.42%
Baseline to 12 months post-randomisation	205	70.21%	104	72.73%
Hospital outpatient services				
6 months post-randomisation	219	75.00%	102	71.33%
12 months post-randomisation	201	68.84%	99	69.23%
Baseline to 12 months post-randomisation	170	58.22%	82	57.34%
General Community-based health services				
6 months post-randomisation	246	84.25%	116	81.12%
12 months post-randomisation	226	77.40%	112	78.32%
Baseline to 12 months post-randomisation	212	72.60%	102	71.33%
Community-based social care services				
6 months post-randomisation	241	82.53%	115	80.42%
12 months post-randomisation	222	76.03%	108	75.52%
Baseline to 12 months post-randomisation	204	69.86%	97	67.83%
Aids and adaptations				
6 months post-randomisation	243	83.22%	113	79.02%
12 months post-randomisation	222	76.03%	112	78.32%
Baseline to 12 months post-randomisation	206	70.55%	99	69.23%
Medications use				
6 months post-randomisation	256	87.67%	124	86.71%
12 months post-randomisation	247	84.59%	123	86.01%
Baseline to 12 months post-randomisation	236	80.82%	116	81.12%
EQ-5D-5L (Participant reported)				
Baseline	292	100.00%	143	100.00%
6 months post-randomisation	256	87.67%	125	87.41%
12 months post-randomisation	252	86.30%	127	88.81%

^a^A cost category is complete if all variables needed to calculate the cost component were available. For example, if patient indicated they used community based social care services but did not provide the number of visits at a particular time point that cost category was considered missing

### Cost of intervention

Mean total intervention costs for all 12 sessions are presented within each group at each site (Additional file 1: Tables S3, S4) (Appendix). These varied between £242 (Site 15 group 1) and £911 (Site 1 group 1). The average costs per group session per participant (including administrative costs) varied from approximately £11.80 (Site 4, group 3) to £67.00 (Site 1, group 1). The mean cost per participant was generally lower across all sites if the target number of participants (n = 6) had been achieved.

### Resource utilization

For health and personal social service use, shown in (Additional file 1: Tables S5, S6), there were non-significant differences between the two groups in utilisation of hospital inpatient and outpatient care, community based health care and social services, and days off work.

### Total economic costs

For the base-case analysis, mean NHS and PSS costs, inclusive of intervention costs, over the entire follow-up period were £1974.06 for the BOOST programme versus £1826.64 for the BPA group (Table 3). There was a non-significant cost difference in favour of the BPA group of £147.42 (95% CI £− 419 to 714).

**Table 3:** NHS and PSS costs by trial allocation arm and cost component category for the entire follow-up period in base case (imputed) analysis (£, 2018–19 prices)

Cost category	Treatment arm, mean; median cost (£) (SE)		Mean cost (£) difference	*p*-value ^i^	95% Confidence interval
	BOOST programme	Best Practice Advice (BPA)			
	(n = 292)	(n = 143)			
NHS and PSS					
Intervention costs	382.31; 374.76 (6.90)	74.61; 70.10 (9.86)	307.71	< 0.0001	(284.04 to 331.37)
Hospital inpatient services	501.69; 0 (86.59)	552.50; 0 (123.74)	− 50.80	0.737	(− 347.67 to 246.07)
Hospital day care services	122.77; 0 (19.65)	91.23; 0 (28.09)	31.54	0.358	(− 35.86 to 98.94)
Hospital outpatient services	407.34; (232.93) (53.98)	495.04; 298.75 (77.14)	− 87.70	0.352	(− 272.78 to 97.38)
General community-based health services	210.02; 172.35 (12.43)	204.59; 179 (17.77)	5.43	0.802	(− 37.19 to 48.06)
Community-based social care services	0.27; 0 (0.86)	5.64; 0 (1.23)	− 5.37	< 0.0001	(− 8.33 to − 2.41)
Equipment, adaptations/repairs	6.02; 0 (1.51)	3.02; 0 (2.16)	3.00	0.256	(− 2.18 to 8.19)
Concomitant/prescription medications	343.63; 280.43 (21.43)	400.02; 347.39(30.62)	− 56.39	0.132	(− 129.86 to 17.08)
Total (NHS and PSS) (excluding intervention costs)	1591.75; 1173 (117.23)	1752.04; 1296 (167.54)	− 160.29	0.434	(− 562.23 to 241.65)
Total (NHS and PSS) (including intervention costs)	1974.06; 1508 (118.05)	1826.64; 1347 (168.70)	147.42	0.474	(− 419.00 to 714.00)

^a^*p*-value calculated using the student’s t-test, two-tail unequal variance

Mean total societal costs, for the entire follow-up period, inclusive of the intervention cost, were £2176.01 in the intervention group compared with £2140.54 in the BPA group (Table 4). This generated a mean cost difference of £35.47 (95% CI: − £469.57 to 540.51) in favour of the BPA group. Societal costs (excluding NHS and PSS costs) were higher in the BPA group and primarily driven by economic valuation of time taken off work by patients and carers in the BPA group. Considering that the mean number of days off work was similar between the two groups and only a relatively small number of patients/carers took time off work, the mean values are skewed by a few individuals (Additional file 1: Figure S1). The estimates of economic costs for non-imputed (complete) cases are shown in Additional file 1: Tables S7, S8 and follow the same pattern as the imputed base case analysis.

**Table 4:** Total societal costs by trial allocation and cost component category for the entire follow-up period in imputed analysis (£, 2018–19 prices)

Cost category	Treatment arm, mean cost (£) (SE)		Mean cost (£) difference	*p*-value i	95% confidence interval
	BOOST programme	Best practice advice (BPA)			
	(n = 292)	(n = 143)			
NHS and PSS (including intervention costs)	1974.06 (118.05)	1826.64 (168.70)	147.42	0.474	(− 419.00 to 714.00)
Societal					
Privately provided health services	14.24 (5.15)	8.32 (7.37)	5.91	0.511	(− 11.76 to 23.59)
Medications	5.53 (0.88)	7.01 (1.26)	− 1.48	0.335	(− 4.49 to 1.53)
Patient equipment	21.17 (8.94)	26.93 (12.78)	− 5.75	0.712	(− 36.41 to 24.91)
Patient travel	13.91 (2.18)	20.86 (3.11)	− 6.96	0.068	(− 14.42 to 0.51)
Time off work	33.50 (29.74)	100.49 (42.50)	− 66.99	0.197	(− 168.95 to 34.96)
Other Societal Costs	113.60 (46.69)	150.29 (66.73)	− 36.68	0.653	(− 196.77 to 123.40)
Societal (excluding NHS and PSS costs)	201.94 (68.80)	313.90 (98.32)	− 111.95	0.351	(− 347.82 to 123.92)
Total Societal (including NHS and PSS and intervention costs)	2176.01 (145.13)	2140.54 (207.40)	35.47	0.889	(− 469.57 to 540.51)

^a^ *p*-value calculated using the student’s t-test, two-tail unequal variance

### Health‑related quality of life outcomes

The adjusted mean (SE) participant-reported QALY estimate for to between 79%-89% across cost-effectiveness thresholds to between 79%-89% across cost-effectiveness thresholds the base case analysis over 12 months favoured the BOOST programme (0.621 (0.009) versus 0.599 (0.006); between group difference 0.021 (95% CI 0 to 0.044)) (Additional file 1: Table S9). These gains were driven by a between group difference in EQ-5D utility scores at 6 months (0.039; [95% CI 0.008 to 0.07]), which narrowed at 12 months (0.009; [95% CI − 0.027 to 0.045]).

### Cost‑effectiveness results: base‑case analysis

**Fig. 1:**
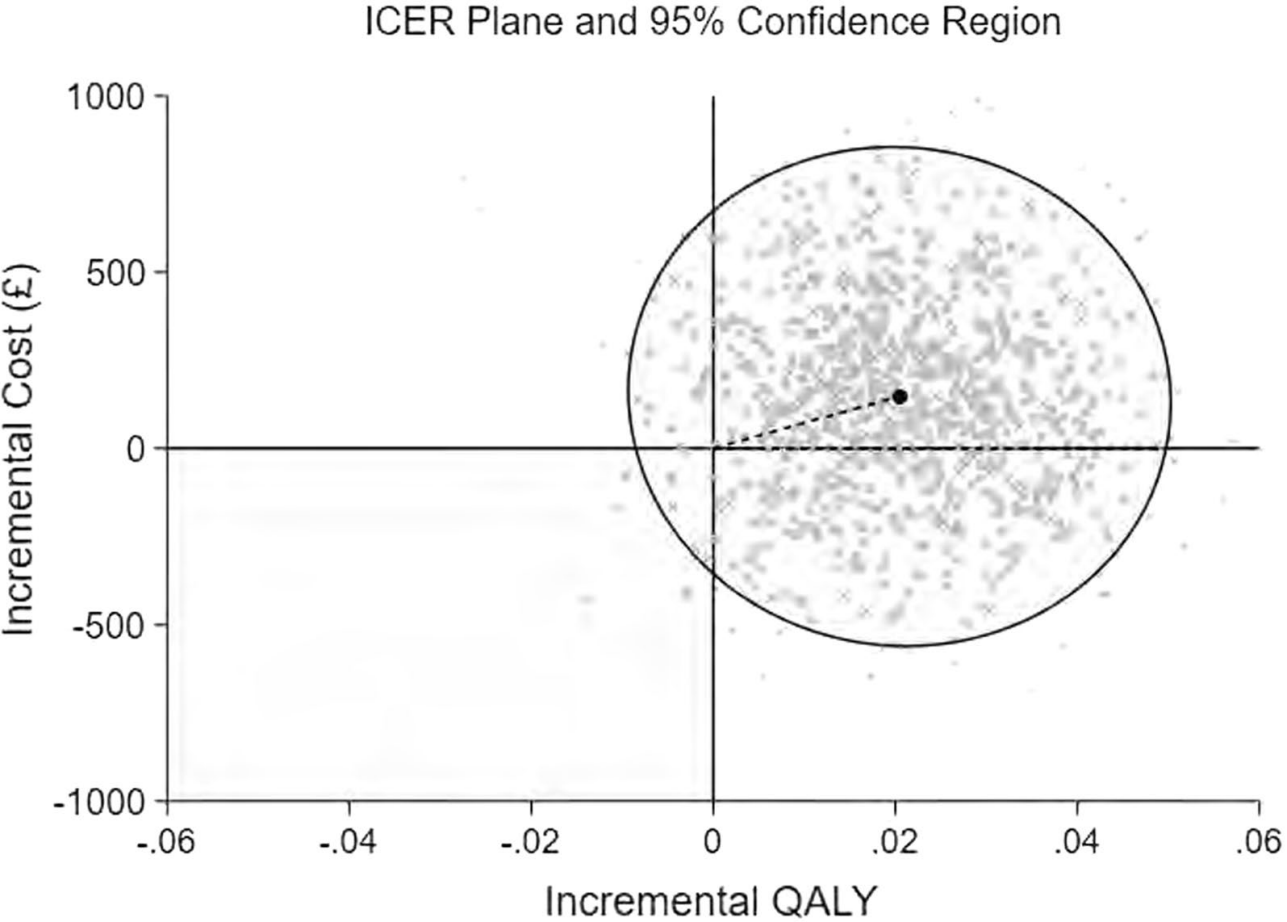
Cost-effectiveness plane for the base case imputed covariate-adjusted analysis

**Fig. 2:**
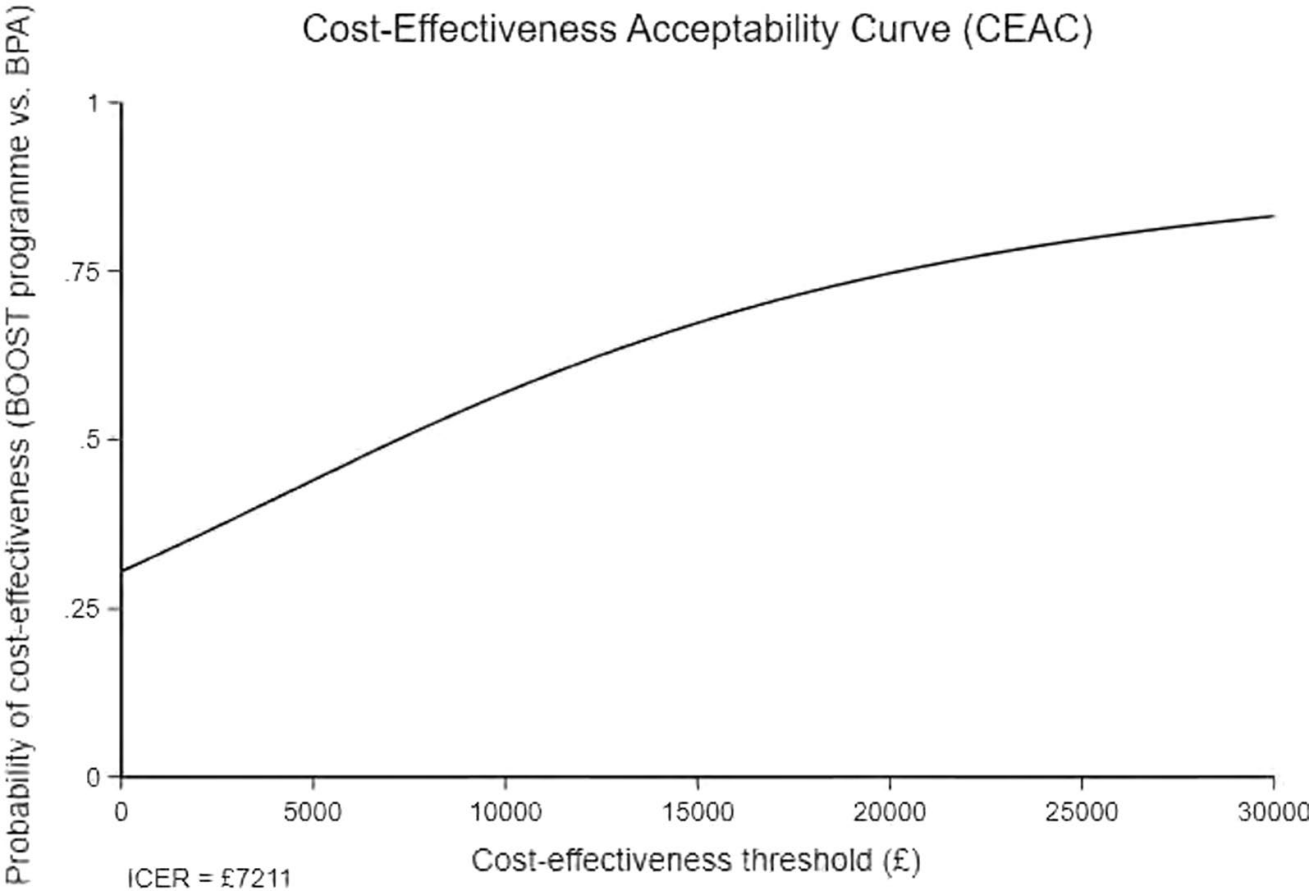
Cost-effectiveness acceptability curve for the base-case imputed (covariate -adjusted) analysis

*NHS and PSS Perspective*. The base-case economic evaluation indicated that the BOOST programme was associated with marginally higher NHS and PSS costs (£147, 95% CI − 419 to 714) and an increase in QALYs (0.020, 95% CI − 0.003 to 0.045). The mean ICER for the BOOST programme was estimated at £7,211 per QALY gained, i.e. on average, the BOOST programme was associated with a higher cost and an increase in QALYs. The associated mean INMB at cost-effectiveness thresholds of £15,000, £20,000 and £30,000 per QALY were £145, £244 and £464, respectively (Table 5). The base-case mean INMB was > 0, suggesting that the BOOST programme would result in an average net economic gain of approximately £244 (INMB = £244, 95% CI − £570 to £1058). The probability of cost-effectiveness for the BOOST programme was estimated as 67%, 78% and 83% at cost-effectiveness thresholds of £15,000, £20,000 and £30,000 per QALY, respectively. The joint distribution of costs and outcomes for the base-case analysis is presented graphically in Fig. 1. The cost-effectiveness acceptability curve is shown in Fig. 2.

**Table 5:** Cost-effectiveness, cost/QALY (£, 2019): BOOST programme compared to Best Practice Advice

Scenario	Mean incremental cost (95% CI)	Mean incremental QALY (95% CI)	ICER^a^	Probability of cost-effectiveness			Net monetary benefits		
	BOOST programme vs. Best Practice Advice	BOOST programme vs. Best Practice Advice		P^1^	P^2^	P^3^	NMB^1^ (95% CI)	NMB^2^ (95% CI)	NMB^3^(95% CI)
Base case (Imputed, covariate adjusted, intention-to-treat) analyses									
NHS and PSS perspective	147.42 (− 419 to 714)	0.020 (− 0.003 to 0.044)	7211	0.673	0.745	0.832	145 (− 600.21 to 890.21)	244 (− 570 to 1058.01)	464 (− 531.90 to 1459.90)
Sensitivity analysis									
Societal perspective	36 (− 470 to 541)	0.021 (− 0.002 to 0.045)	1745	0.788	0.836	0.886	208 (− 534.17 to 950.17)	322 (− 521.72 to 1165.72)	551 (− 522.92 to 1624.92)
Complete case (non-imputed) attributable costs and QALYs, covariate adjusted	182 (− 395 to 759)	0.04 (0.009 to 0.070)	4610	0.851	0.91	0.959	407 (− 374.37 to 1188.37)	605 (− 287.15 to 1497.15)	1000 (− 144.03 to 2144.03)
Subgroup analyses									
Age									
65–74 years	− 6 (− 760 to 747)	0.030 (− 0.003 to 0.063)	Dominant	0.830	0.873	0.915	445 (− 501 to 1391)	595 (− 459 to 1649)	895 (− 413 to 2202)
+ 75 years	331 (− 529 to 1190)	0.010 (− 0.024 to 0.045)	32252	0.371	0.416	0.487	− 183 (− 1243 to 877)	− 132 (− 1302 to 1038)	− 30 (− 1459 to 1399)
Gender									
Male	− 314 (− 1201 to 572)	0.045 (0.009 to 0.080)	Dominant	0.964	0.977	0.987	978 (− 103 to 2058)	1201 (8 to 2393)	1646 (188 to 3103)
Female	491 (− 245 to 1227)	0.002 (− 0.030 to 0.035)	201660	0.167	0.199	0.260	− 467 (− 1398 to 464)	− 455 (− 1492 to 582)	− 431 (− 1716 to 854)
Tilburg frailty indicator									
Not Frail	282.11 (− 539 to 1103)	0.015 (− 0.020 to 0.050)	18769	0.456	0.513	0.594	− 58 (− 1075 to 960)	17 (− 598 to 1044)	168 (− 1231 to 1566)
Frail	− 6 (− 809to 796)	0.020 (− 0.014 to 0.053)	Dominant	0.725	0.763	0.808	293.28 (− 700 to 1286)	391 (− 2129 to 2209)	587 (− 763 to 1937)
ODI cut-off									
ODI < = 22	− 383 (− 1530 to 764)	0.042 (− 0.004 to 0.088)	Dominant	0.926	0.943	0.960	1004 (− 382 to 2390)	1214 (− 313 to 2740)	1634 (− 228 to 3496)
ODI > 22	334 (− 327 to 994)	0.018 (− 0.009 to 0.045)	18677	0.437	0.521	0.640	− 78 (− 902 to 746)	11 (− 901 to 923)	189 (− 930 to 1308)
Fear avoidance belief score									
0–14	346 (− 388 to 1079)	0.019 (− 0.012 to 0.050)	17877	0.452	0.532	0.645	− 73 (− 983 to 837)	23 (− 986 to 1033)	216 (− 1028 to 1460)
15 +	− 135 (− 1039 to 769)	0.024 (− 0.014 to 0.061)	Dominant	0.806	0.834	0.863	492 (− 628 to 1613)	611 (− 631 to 1852)	847 (− 679 to 2374)
Hand grip strength									
Men: < 30; Women: < 20	191 (− 738 to 1119)	0.022 (− 0.015 to 0.059)	8609	0.597	0.655	0.731	136 (− 998 to 1271)	247 (− 1001 to 1496)	468 (− 1051 to 1987)
Men > = 30; Women > = 20	126 (− 628 to 881)	0.020 (− 0.012 to 0.051)	6404	0.642	0.698	0.768	164 (− 759 to 1087)	263 (− 759to 1284)	460 (− 797 to 1717)

P^1^, P^2^, P^3^: probability cost-effective if cost-effectiveness threshold set at £15,000/QALY, £20,000/QALY or £30,000/QALY, respectively

NMB^1^, NMB^2^,NMB^3^: net monetary benefit if cost-effectiveness threshold set at £15,000/QALY, £20,000/QALY or £30,000/QALY, respectively

^a^ICER: Incremental cost-effectiveness ratio. Dominance indicates average costs were less and average benefit greater for BOOST programme vs. BPA

### Sensitivity and subgroup analyses

The sensitivity analysis conducted from a societal perspective increased the probability that the BOOST programme is cost-effective to between 79 and 89% across cost-effectiveness thresholds (Table 5). The sensitivity analysis based on complete cases supported the base-case finding that the BOOST programme was associated with higher costs and increase in QALYs (Table 5). The pre-planned subgroup analyses showed evidence that the BOOST programme was more cost-effective in the following subgroups: males, participants aged 64–74 years, participants with a baseline ODI <  = 22, participants with higher HGs (> = 30 in men; >  = 20 women),participants with higher fear avoidance, and participants classified as frail based on Tilburg Frailty Index (TFI < 5).

## Discussion

This trial-based economic evaluation revealed that the BOOST programme led, on average, to a modest increase in health-related quality of life, at a small increased cost, over a 12 month period. The resulting ICER from an NHSS and PSS perspective of £7,211 per QALY gained falls favourably below the recommended NICE cost-effectiveness threshold of £20,000 per QALY though the uncertainty around the mean ICER was large. From a societal perspective, the BOOST programme was more cost-effective with a mean ICER of £1745 per QALY gained.

We undertook a robust analysis, based in general on good completion of follow up questionnaires. We used two perspectives to estimate cost-effectiveness. The NHS and PSS perspective adopted within the base-case analysis considered all costs related to providing health and related care. The societal perspective also considered economic values of work absences by the trial participants and their informal carers; the latter is often a vital lifeline to older people with NC. Complete case and imputed analyses were broadly consistent in outcomes. Imputed analyses gave a more conservative estimate, most likely due to greater numbers of frail, older women dropping out of follow up. The evidence of HRQoL benefits add to the emerging evidence base from good quality clinical trials that demonstrate improvements in mobility from similar programmes [3]. In additional to improvements in HRQoL, the BOOST programme also resulted in improvements in mobility over a 12 month period and improvements in pain related disability over a 6 month period [22].

Without economic modelling beyond the current parameters of the trial, the longer-term cost-effectiveness of the BOOST programme cannot be ascertained. However, the pattern of rising and then declining HRQoL gains over the year-long follow-up period suggest that it is unlikely that, without further intervention the effects will be sustained over a longer time-horizon. Recent evidence from the REACT study showed that long-term exercise programmes (i.e., those lasting at least 1 year) can improve physical functioning among older adults in real-world community settings in the UK, with benefits that are sustained for at least 24 months [23]. The group-based exercises in the REACT study were delivered over the entire 12 month period compared to the group-based component of the BOOST programme which lasted 12 weeks (with participants continuing exercises independently up to 12 months). Delivery of the group-based component of the BOOST programme over a longer period and assessment of economic outcomes over a longer follow-up period may therefore have generated a different pattern of results.

We noted that the intervention appears less cost-effective in participants who were older and more disabled by pain and in females. These participants may have greater challenges with attending and complying with the intervention. Compliance with the group intervention, defined as attending at least 9 out of 12 group sessions, seemed to be one of the key factors driving the effectiveness of the BOOST programme. The difference in the main outcome measure was larger, favoured the BOOST programme and reached the predefined clinically significant threshold (5 points on the ODI) at 6 months when compliance with group intervention was considered. As with all sub-group analyses, these should be considered exploratory only, and our primary estimates account for all people regardless of their compliance to the intervention and follow up. We used a pragmatic approach to sampling, and hence our findings should be generalisable. We limited entry to the trial to those greater than 65 years. Neurogenic claudication is generally associated with ageing, and incidence below 65 years declines.

There is limited evidence for cost-effectiveness of structured and progressive exercises for patients with NC in the broader literature. The cost-effectiveness studies that have been conducted in this population group have focussed on cost-effectiveness of surgical treatment of spinal stenosis (in patients with symptoms of NC) [24], or assessed physiotherapy as part of a ‘conservative care’ package (alongside pain medications and epidural injections) [25]. Two recently published RCTs examined programmes similar to the BOOST programme [3, 4]. Both studies assessed HRQoL via the SF-36 health-related quality of life questionnaire and neither reported costs associated with the structured exercise programmes or their downstream resource consequences. To the best of our knowledge, this economic evaluation is based on the largest RCT of its kind reported to date.

Strengths of the current economic evaluation are that the trial was prospectively designed for a cost-effectiveness analysis using individual-level data to reach a confirmatory conclusion regarding a physiotherapy-delivered physical and psychological group intervention in older adults with NC. A rigorous evaluation of the costs associated with the delivery of the BOOST programme is presented, based on prospective (observed) data on the time it took staff to run the group sessions, administrative costs, equipment costs and follow-up costs. In a non-trial (real) setting, costs of delivering the intervention may be lower as there will be less administrative burden of completing treatment logs and hence less time needed to run the groups.

There are some limitations to this economic evaluation. Firstly, utility measurements were collected at only two time-points post-randomisation. Evidence suggests that the timing of assessment can significantly influence cost-effectiveness results when using the EQ-5D, particularly when participants experience recurrent health fluctuations [26]. In such cases, the linear interpolation of utility data may fail to reflect HRQoL fluctuations over short periods and the uncertainty is compounded by missing data. Secondly, resource use data were retrospectively recalled by participants, and this could have led to recall bias, though we cannot predict the direction of this bias. Findings form literature are mixed, suggesting that resource use may be under-reported, over-reported or they may be good agreement between patient/carer recall and data extracted from medical records, depending on how well the resource use measures are structured [27]. Because the recall periods and questionnaires were standardised across randomised groups, retrospective recall is unlikely to have biased results in favour of one group. Thirdly, our approaches to collecting resource use data did not disentangle resource use associated with NC from resource use associated with broader health factors. Fourthly, constrained trial resources precluded an assessment of the health-related quality of life outcomes of carers and therefore these potential externalities were excluded from the sensitivity analysis conducted from a societal perspective.

## Conclusion

The BOOST programme resulted in modest QALY gains over a short-term (12 month) follow-up period. Future studies with longer intervention and follow-up periods are needed to address uncertainty around the health-related quality of life impacts and cost-effectiveness of such programmes.

## Supplementary Information

**Additional file 1: Table S1.** Unit costs for broader resource items (£, 2018–19). **Table S2.** Unit costs of standard materials used to deliver intervention. **Table S3.** Total cost of delivering intervention by site and group. **Table S4.** Mean staff cost (£, 2019) per session per participant. **Table S5.** Health resource use by trial allocation, category and study period for complete cases at 6months post-randomisation. **Table S6.** Health resource use by trial allocation, category and study period for complete cases at 12 months post-randomisation. **Table S7.** Economic costs by trial allocation arm and cost component category for the entire follow-up period for the NHS PSS perspective among complete cases (£, 2018–19 prices). **Table S8.** Economic costs by trial allocation arm and cost component category for the entire follow-up period for the societal perspective among complete cases (£, 2018–19 prices). **Table S9.** Patient reported EQ-5D-5L utility scores and QALYs (Imputed Analysis). **Figure S1.** Economic losses due to lost days of work by participants and/carers (£, 2018–19)

## Data Availability

The datasets analysed during the current study are available from the corresponding author upon reasonable request.

## Abbreviations

BOOST: Better outcomes for older people with spinal trouble
BPA: Best practice advice
CIs: Confidence intervals
FAB: Fear avoidance beliefs questionnaire
HGS: Hand grip strength
HRQoL: Health-related quality of life
ICER: Incremental cost-effectiveness ratio
INMB: Incremental net monetary benefit
MAR: Missing at random
NC: Neurogenic claudication
NHS: National health service
NHSCII: NHS cost inflation index
NICE: National institute for health and care excellence
ODI: Oswestry disability index
PSS: Personal social services
PSSRU: Personal social services research unit
QALY: Quality-adjusted life year
RCT: Randomised controlled trial
SE: Standard error
SUR: Seemingly unrelated regression
TFI: Tilburg frailty index
