# Correction to: Economic costs, health-related quality of life outcomes and cost-utility of a physical and psychological group intervention targeted at older adults with neurogenic claudication

**DOI:** 10.1186/s12962-023-00431-1

**Published:** 2023-04-11

**Authors:** Mandy Maredza, Kamran Khan, Ioana R. Marian, Susan J. Dutton, Esther Williamson, Sarah E. Lamb, Stavros Petrou

**Affiliations:** 1 Division of Health Sciences, Warwick Medical School, The University of Warwick, Coventry, UK; 2 Warwick Clinical Trials Unit, University of Warwick, Warwick, UK; 3 Nuffield Department of Orthopaedics, Rheumatology and Musculoskeletal Sciences, Oxford Clinical Trials Research Unit, University of Oxford, Oxford, UK; 4 Exeter Medical School, University of Exeter, Devon, UK; 5 Nuffield Department of Primary Care Health Sciences, University of Oxford, Oxford, UK

**Cost Effectiveness and Resource Allocation (2023) 21:14**

10.1186/s12962-022-00410-y

Following publication of the original article [1], it came to the authors’ attention that the names of the authors without middle names had been erroneously switched around (e.g., Mandy Maredza had been published as Maredza Mandy). The names have been corrected in the published article and the corrected names can be seen in the author list of this erratum.

