# Supplementary material for: Economic costs, health-related quality of life outcomes and cost-utility of a physical and psychological group intervention targeted at older adults with neurogenic claudication
Source: Cost Eff Resour Alloc. 2023 Feb 8;21:14. doi: 10.1186/s12962-022-00410-y (PMC9906820; doi:10.1186/s12962-022-00410-y)
Supplement: Supplementary file 1 — Additional file 1: Table S1. Unit costs for broader resource items (£, 2018–19). Table S2. Unit costs of standard materials used to deliver intervention. Table S3. Total cost of delivering intervention by site and group. Table S4. Mean staff cost (£, 2019) per session per participant. Table S5. Health resource use by trial allocation, category and study period for complete cases at 6months post-randomisation. Table S6. Health resource use by trial allocation, category and study period for complete cases at 12 months post-randomisation. Table S7. Economic costs by trial allocation arm and cost component category for the entire follow-up period for the NHS PSS perspective among complete cases (£, 2018–19 prices). Table S8. Economic costs by trial allocation arm and cost component category for the entire follow-up period for the societal perspective among complete cases (£, 2018–19 prices). Table S9. Patient reported EQ-5D-5L utility scores and QALYs (Imputed Analysis). Figure S1. Economic losses due to lost days of work by participants and/carers (£, 2018–19) [file 12962_2022_410_MOESM1_ESM.docx]

**Additional file Digital Content**

**Economic costs, health-related quality of life outcomes and cost-effectiveness of a physical and psychological group intervention targeted at older adults with neurogenic claudication**

**Table S1 Unit costs for broader resource items (£, 2018-19)**

| **Cost variable** | **Unit Cost^[[1]](#endnote-1)^** | **Unit of analysis** | **Source** |
| --- | --- | --- | --- |
| **Hospital Outpatient Services** |  |  |  |
| Orthopaedic/spinal clinic | 128 | Per contact | NHS Reference Costs 2018 [1] |
| Physiotherapy department | 55 | Per contact | NHS Reference Costs 2018 [1] |
| Accident & Emergency | 136 | Per contact | NHS Reference Costs 2018 [1] |
| Geriatrician | 257 | Per contact | NHS Reference Costs 2018 [1] |
|  |  |  |  |
| **General community-based health services** |  |  |  |
| General practitioner –Office visit | 33 | per office consultation lasting 9.22 min | PSSRU 2019 [2] |
| General practitioner –Home visit | 3.579176 | per minute | PSSRU 2015 [3] |
| Practice nurse –Office visit | 37 | per hour | PSSRU 2019 [2] |
| Practice nurse –Home visit | 37 | per hour | PSSRU 2019 [2] |
| District nurse –Office visit | 31 | per hour | PSSRU 2010 [4] |
| District nurse –Home visit | 68 | per hour | PSSRU 2010 [4] |
| Health visitor– Office Visit | 31 | per hour | PSSRU 2010 [4] |
| Health visitor– Home Visit | 107 | per hour | PSSRU 2010 [4] |
| Incontinence nurse – Office visit | 22 | Per surgery consultation | PSSRU 2015 [3] |
| Incontinence nurse – Home Visit | 33.75 | Per home visit lasting 25min | PSSRU 2015 [3] |
| Occupational therapist – Office visit | 47 | Per hour | PSSRU 2019 [2] |
| Occupational therapist – Home Visit | 44 | per hour | PSSRU 2019 [2] |
| Physiotherapist – Office visit | 47 | per hour | PSSRU 2019 [2] |
| Community chiropodist | 32 | per hour | PSSRU 2014 [5] |
| **Social care services** |  |  |  |
| Care manager | 40 | per hour | PSSRU 2019 [2] |
| Social worker | 45 | per hour | PSSRU 2019 [2] |
| Home care worker | 28 | per weekday hour | PSSRU 2019 [2] |
| Sitting scheme – Home Visit | 6.5 | per hour | <https://www.ageuk.org.uk/northamptonshire/our-services/carers-sitting-service/> |
| Meals on wheels | 3.6 | per meal | <https://www.publicsectorcatering.co.uk/sites/default/files/attachment/nacc_-_meals_on_wheels_report_2018.pdf> |
| Laundry service | 4.9 | per load | <https://www.northyorks.gov.uk/paying-care-home> |
| **Productivity losses (Participant and carers)** |  |  |  |
| Days off work ^[[2]](#endnote-2)^ | Varied | Mean Weekly earning | <https://www.ons.gov.uk/employmentandlabourmarket/peopleinwork/earningsandworkinghours/bulletins/annualsurveyofhoursandearnings/2019#employee-earnings-and-hours-worked> |

**Table S2 Unit costs of standard materials used to deliver intervention**

| **Item** | **Cost per item (£, 2018-19)** | **Source of unit cost** |
| --- | --- | --- |
| Lap counter | £2.24 | Trial expenditure records |
| Stopwatch | £5.31 | NHS Supply Chain Catalogue 2018 [6] |
| 1 pair 1.5kg ankle weights | £26.38 | Trial expenditure records |
| 1 pair 3.0kg ankle weights | £38.64 | Trial expenditure records |
| 1 pair 5kg ankle weights | £54.08 | Trial expenditure records |
| 1 set of dubmbells + stand (1.5, 3kg, 5kg) | £47.33 | Trial expenditure records |
| Small weighted vest (20lb/ 9kg) | £76.04 | [https://www.physioparts.co.uk/high-performance-weight-vest-9-kg-18-kg (accessed 15 Nov 2019)](https://www.physioparts.co.uk/high-performance-weight-vest-9-kg-18-kg%20(accessed%2015%20Nov%202019)) |
| large weights vest (40lb/18kg) | £99.65 | <https://www.physioparts.co.uk/muscle-power-gewichtsvest-20-kg?dfw_tracker=16973-FS14944GB&gclid=Cj0KCQiAtrnuBRDXARIsABiN-7AZOdPtkH7b10h621uO8BPG9sh5q0tdutcr2bxpvpXdz7WkM_ZBiyMaArHiEALw_wcB> |
| 1 x small weights belt (12kg) | £128.00 | [https://www.mikesdivestore.com/products/scubapro-soft-lead-weights?variant=384641316  https://www.mikesdivestore.com/products/scubapro-padded-weight-belt?variant=968758189&cmp_id=9560734000&adg_id=100704686569&kwd=&device=c&gclid=CjwKCAjw4871BRAjEiwAbxXi2_5FfQdPtjBs1Ti5L7qFl0_Nl_TFaKiAiEmZQROaX6LFyALzmMIu5hoCVS0QAvD_BwE](https://www.mikesdivestore.com/products/scubapro-soft-lead-weights?variant=384641316) |
| Wheeled trolley | £20.08 | Trial expenditure records |
| *Home use ankle weights* |  |  |
| 0.5kg pair | £4.35 | NHS Supply Chain Catalogue |
| 1 kg pair | £4.35 | NHS Supply Chain Catalogue |
| 1.5kg pair | £10 | [Gallant Wrist and Ankle Weights \| Arm and leg weights – Gallant Sport (gallantsports.co.uk)](https://www.gallantsports.co.uk/products/gallant-wrist-ankle-weights)  Last accessed 5 October 2022 |
| 2.5kg pair | £14.99 | [Gallant Wrist and Ankle Weights \| Arm and leg weights – Gallant Sport (gallantsports.co.uk)](https://www.gallantsports.co.uk/products/gallant-wrist-ankle-weights)  Last accessed 5 October 2022 |

**Table S3 Total cost of delivering intervention by site and group**

| **Site Code** | **Group** | **Participants per group** | **Total number of sessions delivered** | **Mean number of sessions attended** | **Total Staff Costs (£, 2019)** | | | **Equipment Costs (£, 2019)^[[3]](#endnote-3)^** | **Total Costs (£, 2019)** | **Mean Total Intervention Costs (£,2019)** |
| --- | --- | --- | --- | --- | --- | --- | --- | --- | --- | --- |
|  |  |  |  |  | **Baseline one-on-one session^[[4]](#endnote-4)^** | **Group sessions ^[[5]](#endnote-5)^** | **Follow-up costs^[[6]](#endnote-6)^** |  |  |  |
| 1 | 1 | 2 | 12 | 12.00 | 135.00 | 1610.00 | 44.80 | 32.80 | 1820.00 | 911.00 |
| 1 | 2 | 5 | 12 | 10.60 | 336.00 | 1800.00 | 89.70 | 82.10 | 2310.00 | 462.00 |
| 1 | 3 | 5 | 10 | 8.40 | 336.00 | 1450.00 | 112.00 | 82.10 | 1980.00 | 396.00 |
| 1 | 4 | 5 | 12 | 9.00 | 336.00 | 1630.00 | 89.70 | 82.10 | 2140.00 | 427.00 |
| 1 | 5 | 7 | 11 | 7.60 | 471.00 | 1730.00 | 135.00 | 115.00 | 2450.00 | 351.00 |
| 1 | 6 | 9 | 11 | 7.70 | 605.00 | 1610.00 | 112.00 | 148.00 | 2480.00 | 275.00 |
|  |  |  |  |  |  |  |  |  |  |  |
| 2 | 1 | 5 | 12 | 9.80 | 275.00 | 2208.00 | 215.00 | 60.10 | 2758.00 | 552.00 |
| 2 | 2 | 7 | 12 | 7.70 | 348.00 | 1243.00 | 228.00 | 84.20 | 1903.00 | 272.00 |
| 2 | 3 | 7 | 12 | 8.70 | 389.00 | 1983.00 | 199.00 | 84.20 | 2655.00 | 379.00 |
| 2 | 4 | 4 | 12 | 11.00 | 228.00 | 1131.00 | 140.00 | 48.10 | 1547.00 | 387.00 |
| 2 | 5 | 7 | 12 | 8.30 | 327.00 | 1595.00 | 280.00 | 84.20 | 2286.00 | 327.00 |
| 2 | 6 | 4 | 12 | 9.50 | 187.00 | 1024.00 | 187.00 | 48.10 | 1446.00 | 361.00 |
|  |  |  |  |  |  |  |  |  |  |  |
| 3 | 1 | 6 | 12 | 10.00 | 280.00 | 1100.00 | 150.00 | 61.20 | 1600.00 | 266.00 |
| 3 | 2 | 6 | 12 | 10.00 | 280.00 | 1500.00 | 150.00 | 61.20 | 2000.00 | 333.00 |
| 3 | 3 | 5 | 12 | 7.00 | 234.00 | 1180.00 | 109.00 | 51.00 | 1580.00 | 316.00 |
| 3 | 4 | 6 | 12 | 8.70 | 280.00 | 1230.00 | 164.00 | 61.20 | 1740.00 | 289.00 |
|  |  |  |  |  |  |  |  |  |  |  |
| 4 | 1 | 6 | 11 | 8.70 | 280.00 | 1410.00 | 150.00 | 93.00 | 1930.00 | 322.00 |
| 4 | 2 | 5 | 11 | 9.60 | 234.00 | 1250.00 | 136.00 | 77.50 | 1690.00 | 339.00 |
| 4 | 3 | 9 | 12 | 10.00 | 421.00 | 1100.00 | 245.00 | 139.00 | 1900.00 | 212.00 |
|  |  |  |  |  |  |  |  |  |  |  |
| 5 | 1 | 3 | 12 | 9.30 | 170.00 | 1710.00 | 99.40 | 42.90 | 2020.00 | 673.00 |
| 5 | 2 | 3 | 12 | 11.00 | 170.00 | 1310.00 | 99.40 | 42.90 | 1620.00 | 541.00 |
| 5 | 3 | 8 | 12 | 9.50 | 454.00 | 2050.00 | 265.00 | 115.00 | 2880.00 | 360.00 |
| 5 | 4 | 4 | 12 | 12.00 | 227.00 | 1390.00 | 133.00 | 57.30 | 1810.00 | 453.00 |
|  |  |  |  |  |  |  |  |  |  |  |
| 7 | 1 | 7 | 12 | 10.00 | 327.00 | 1760.00 | 123.00 | 133.00 | 2340.00 | 335.00 |
| 7 | 2 | 3 | 12 | 7.00 | 140.00 | 1230.00 | 54.50 | 56.90 | 1480.00 | 495.00 |
| 7 | 3 | 4 | 10 | 6.30 | 187.00 | 1260.00 | 27.30 | 75.90 | 1560.00 | 389.00 |
| 7 | 4 | 7 | 12 | 8.60 | 327.00 | 1190.00 | 95.40 | 133.00 | 1750.00 | 250.00 |
|  |  |  |  |  |  |  |  |  |  |  |
| 8 | 1 | 6 | 12 | 9.50 | 280.00 | 1590.00 | 123.00 | 93.70 | 2090.00 | 348.00 |
| 8 | 2 | 7 | 12 | 8.30 | 327.00 | 2560.00 | 109.00 | 109.00 | 3100.00 | 443.00 |
| 8 | 3 | 7 | 12 | 8.30 | 327.00 | 1990.00 | 95.40 | 109.00 | 2520.00 | 361.00 |
|  |  |  |  |  |  |  |  |  |  |  |
| 9 | 1 | 4 | 12 | 9.80 | 217.00 | 1850.00 | 104.00 | 61.90 | 2230.00 | 558.00 |
| 9 | 2 | 4 | 12 | 9.00 | 227.00 | 1810.00 | 94.90 | 61.90 | 2190.00 | 548.00 |
| 9 | 3 | 4 | 11 | 9.80 | 197.00 | 1690.00 | 91.90 | 61.90 | 2040.00 | 510.00 |
| 9 | 4 | 4 | 12 | 8.80 | 197.00 | 1690.00 | 104.00 | 61.90 | 2050.00 | 514.00 |
| 9 | 5 | 4 | 12 | 11.00 | 197.00 | 1490.00 | 66.30 | 61.90 | 1810.00 | 454.00 |
|  |  |  |  |  |  |  |  |  |  |  |
| 10 | 1 | 3 | 12 | 11.00 | 140.00 | 1170.00 | 81.80 | 48.90 | 1440.00 | 479.00 |
| 10 | 2 | 5 | 12 | 11.00 | 234.00 | 1160.00 | 109.00 | 81.50 | 1580.00 | 316.00 |
| 10 | 3 | 10 | 12 | 8.50 | 467.00 | 1900.00 | 204.00 | 163.00 | 2730.00 | 273.00 |
|  |  |  |  |  |  |  |  |  |  |  |
| 11 | 1 | 6 | 12 | 9.80 | 341.00 | 1760.00 | 94.60 | 84.80 | 2280.00 | 379.00 |
| 11 | 2 | 4 | 12 | 9.00 | 238.00 | 1190.00 | 58.40 | 56.50 | 1540.00 | 386.00 |
| 11 | 3 | 6 | 12 | 11.00 | 341.00 | 1910.00 | 106.00 | 84.80 | 2440.00 | 406.00 |
| 11 | 4 | 6 | 12 | 8.70 | 404.00 | 1230.00 | 154.00 | 84.80 | 1870.00 | 312.00 |
|  |  |  |  |  |  |  |  |  |  |  |
| 12 | 1 | 4 | 12 | 11.00 | 187.00 | 1900.00 | 125.00 | 115.00 | 2320.00 | 581.00 |
| 12 | 2 | 2 | 12 | 10.00 | 93.50 | 1280.00 | 62.30 | 57.40 | 1490.00 | 746.00 |
| 12 | 3 | 4 | 11 | 9.50 | 187.00 | 1870.00 | 125.00 | 115.00 | 2290.00 | 573.00 |
| 12 | 4 | 4 | 12 | 6.80 | 187.00 | 1270.00 | 125.00 | 115.00 | 1700.00 | 425.00 |
|  |  |  |  |  |  |  |  |  |  |  |
| 13 | 1 | 6 | 12 | 9.00 | 275.00 | 1350.00 | 133.00 | 140.00 | 1890.00 | 316.00 |
| 13 | 2 | 3 | 12 | 7.30 | 170.00 | 1220.00 | 46.80 | 69.90 | 1510.00 | 503.00 |
|  |  |  |  |  |  |  |  |  |  |  |
| 14 | 1 | 4 | 11 | 8.00 | 187.00 | 1490.00 | 125.00 | 68.70 | 1870.00 | 466.00 |
| 14 | 2 | 5 | 12 | 11.00 | 234.00 | 1880.00 | 156.00 | 85.90 | 2360.00 | 471.00 |
| 14 | 3 | 4 | 12 | 9.00 | 187.00 | 1490.00 | 109.00 | 68.70 | 1850.00 | 463.00 |
|  |  |  |  |  |  |  |  |  |  |  |
| 15 | 1 | 8 | 12 | 8.60 | 350.00 | 1320.00 | 119.00 | 145.00 | 1940.00 | 242.00 |
| 15 | 2 | 5 | 12 | 11.00 | 234.00 | 968.00 | 95.40 | 90.70 | 1390.00 | 278.00 |

**Table S4 Mean staff cost (£, 2019) per session per participant**

|  |  |  |  |  |  |  |  | **Sensitivity analyses, mean cost (£) per session per participant^[[7]](#endnote-7)^** | | |
| --- | --- | --- | --- | --- | --- | --- | --- | --- | --- | --- |
| **Site Code** | **Group No.** | **Participants per group** | **Total number of sessions delivered** | **Mean number of sessions attended** | **Mean staff cost (£, 2019) per session per participant^[[8]](#endnote-8)^** | **Mean administration costs (£, 2019) per session^[[9]](#endnote-9)^** | **Mean cost (£, 2019) per session per participant (including administrative costs)^[[10]](#endnote-10)^** | **Participants per group** | | |
|  |  |  |  |  |  |  |  | **(n=2)** | **(n=6)** | **(n=10)** |
| 1 | 1 | 2 | 12 | 12.00 | 56.30 | 10.80 | 67.00 | 56.30 | 18.76 | 11.26 |
| 1 | 2 | 5 | 12 | 11.00 | 29.40 | 4.60 | 34.00 | 64.95 | 21.65 | 12.99 |
| 1 | 3 | 5 | 10 | 8.40 | 29.90 | 4.60 | 34.50 | 29.90 | 29.91 | 29.91 |
| 1 | 4 | 5 | 12 | 9.00 | 30.30 | 5.92 | 36.20 | 56.73 | 18.91 | 11.35 |
| 1 | 5 | 7 | 11 | 7.60 | 28.80 | 3.91 | 32.70 | 63.97 | 21.32 | 12.79 |
| 1 | 6 | 9 | 11 | 7.70 | 20.10 | 3.24 | 23.30 | 56.93 | 18.98 | 11.39 |
|  |  |  |  |  |  |  |  |  |  |  |
| 2 | 1 | 5 | 12 | 9.80 | 37.70 | 7.36 | 45.10 | 78.52 | 26.17 | 15.70 |
| 2 | 2 | 7 | 12 | 7.70 | 19.30 | 3.70 | 23.00 | 63.80 | 21.27 | 12.76 |
| 2 | 3 | 7 | 12 | 8.70 | 25.10 | 7.43 | 32.50 | 82.72 | 27.57 | 16.54 |
| 2 | 4 | 4 | 12 | 11.00 | 21.90 | 3.23 | 25.10 | 50.53 | 16.84 | 10.11 |
| 2 | 5 | 7 | 12 | 8.30 | 24.40 | 3.12 | 27.50 | 70.08 | 23.36 | 14.02 |
| 2 | 6 | 4 | 12 | 9.50 | 22.70 | 4.21 | 27.00 | 46.27 | 15.42 | 9.25 |
|  |  |  |  |  |  |  |  |  |  |  |
| 3 | 1 | 6 | 12 | 10.00 | 15.60 | 2.76 | 18.40 | 39.76 | 13.25 | 7.95 |
| 3 | 2 | 6 | 12 | 10.00 | 21.40 | 2.90 | 24.30 | 55.21 | 18.40 | 11.04 |
| 3 | 3 | 5 | 12 | 7.00 | 31.40 | 2.46 | 33.80 | 54.89 | 18.30 | 10.98 |
| 3 | 4 | 6 | 12 | 8.70 | 20.80 | 2.89 | 23.70 | 36.35 | 12.12 | 7.27 |
|  |  |  |  |  |  |  |  |  |  |  |
| 4 | 1 | 6 | 11 | 8.70 | 21.40 | 5.70 | 27.10 | 47.17 | 15.72 | 9.43 |
| 4 | 2 | 5 | 11 | 9.60 | 19.60 | 6.39 | 26.00 | 38.36 | 12.79 | 7.67 |
| 4 | 3 | 9 | 12 | 10.00 | 9.26 | 2.55 | 11.80 | 37.05 | 12.35 | 7.41 |
|  |  |  |  |  |  |  |  |  |  |  |
| 5 | 1 | 3 | 12 | 9.30 | 40.70 | 20.20 | 60.90 | 47.52 | 15.84 | 9.50 |
| 5 | 2 | 3 | 12 | 11.00 | 28.40 | 11.40 | 39.80 | 40.22 | 13.41 | 8.04 |
| 5 | 3 | 8 | 12 | 9.50 | 21.70 | 5.27 | 26.90 | 68.57 | 22.86 | 13.71 |
| 5 | 4 | 4 | 12 | 12.00 | 22.50 | 7.21 | 29.70 | 43.97 | 14.66 | 8.79 |
|  |  |  |  |  |  |  |  |  |  |  |
| 7 | 1 | 7 | 12 | 10.00 | 17.60 | 6.52 | 24.10 | 54.30 | 18.10 | 10.86 |
| 7 | 2 | 3 | 12 | 7.00 | 38.90 | 19.80 | 58.70 | 34.02 | 11.34 | 6.80 |
| 7 | 3 | 4 | 10 | 6.30 | 36.00 | 14.60 | 50.60 | 39.62 | 13.21 | 7.92 |
| 7 | 4 | 7 | 12 | 8.60 | 13.70 | 6.24 | 19.90 | 34.70 | 11.57 | 6.94 |
|  |  |  |  |  |  |  |  |  |  |  |
| 8 | 1 | 6 | 12 | 9.50 | 23.30 | 4.63 | 27.90 | 56.28 | 18.76 | 11.26 |
| 8 | 2 | 7 | 12 | 8.30 | 38.90 | 5.14 | 44.10 | 94.10 | 31.37 | 18.82 |
| 8 | 3 | 7 | 12 | 8.30 | 29.10 | 5.28 | 34.40 | 70.27 | 23.42 | 14.05 |
|  |  |  |  |  |  |  |  |  |  |  |
| 9 | 1 | 4 | 12 | 9.80 | 35.00 | 12.40 | 47.40 | 65.31 | 16.93 | 9.58 |
| 9 | 2 | 4 | 12 | 9.00 | 40.40 | 9.89 | 50.30 | 60.55 | 20.18 | 12.11 |
| 9 | 3 | 4 | 11 | 9.80 | 34.50 | 8.79 | 43.30 | 56.12 | 18.71 | 11.22 |
| 9 | 4 | 4 | 12 | 8.80 | 38.70 | 9.60 | 48.30 | 56.48 | 18.83 | 11.30 |
| 9 | 5 | 4 | 12 | 11.00 | 27.40 | 5.72 | 33.10 | 51.34 | 17.11 | 10.27 |
|  |  |  |  |  |  |  |  |  |  |  |
| 10 | 1 | 3 | 12 | 11.00 | 26.30 | 10.20 | 36.50 | 35.05 | 11.68 | 7.01 |
| 10 | 2 | 5 | 12 | 11.00 | 15.30 | 6.51 | 21.80 | 35.05 | 11.68 | 7.01 |
| 10 | 3 | 10 | 12 | 8.50 | 17.20 | 5.12 | 22.30 | 60.83 | 20.28 | 12.17 |
|  |  |  |  |  |  |  |  |  |  |  |
| 11 | 1 | 6 | 12 | 9.80 | 19.10 | 10.60 | 29.80 | 47.05 | 15.68 | 9.41 |
| 11 | 2 | 4 | 12 | 9.00 | 23.40 | 9.74 | 33.10 | 35.05 | 11.68 | 7.01 |
| 11 | 3 | 6 | 12 | 11.00 | 25.60 | 4.13 | 29.80 | 68.38 | 22.79 | 13.68 |
| 11 | 4 | 6 | 12 | 8.70 | 19.40 | 4.22 | 23.60 | 42.79 | 14.26 | 8.56 |
|  |  |  |  |  |  |  |  |  |  |  |
| 12 | 1 | 4 | 12 | 11.00 | 29.20 | 15.90 | 45.20 | 51.15 | 17.05 | 10.23 |
| 12 | 2 | 2 | 12 | 10.00 | 48.20 | 15.80 | 63.90 | 40.15 | 13.38 | 8.03 |
| 12 | 3 | 4 | 11 | 9.50 | 30.70 | 18.40 | 49.10 | 48.61 | 16.20 | 9.72 |
| 12 | 4 | 4 | 12 | 6.80 | 35.00 | 12.10 | 47.20 | 40.86 | 13.62 | 8.17 |
|  |  |  |  |  |  |  |  |  |  |  |
| 13 | 1 | 6 | 12 | 9.00 | 19.30 | 5.64 | 24.90 | 43.42 | 14.47 | 8.68 |
| 13 | 2 | 3 | 12 | 7.30 | 42.60 | 12.90 | 55.50 | 39.04 | 13.01 | 7.81 |
|  |  |  |  |  |  |  |  |  |  |  |
| 14 | 1 | 4 | 11 | 8.00 | 35.90 | 10.50 | 46.40 | 47.87 | 15.96 | 9.57 |
| 14 | 2 | 5 | 12 | 11.00 | 28.80 | 6.01 | 34.80 | 64.83 | 21.61 | 12.97 |
| 14 | 3 | 4 | 12 | 9.00 | 32.00 | 9.34 | 41.40 | 48.02 | 16.01 | 9.60 |
|  |  |  |  |  |  |  |  |  |  |  |
| 15 | 1 | 8 | 12 | 8.60 | 16.90 | 2.24 | 19.20 | 48.69 | 16.23 | 9.74 |
| 15 | 2 | 5 | 12 | 11.00 | 15.30 | 2.64 | 17.90 | 34.40 | 11.47 | 6.88 |

**Table S5 Health resource use by trial allocation, category and study period for complete cases at 6months post-randomisation**

| **Resource Category (unit)** | **Treatment arm** | | | | | **P value** |
| --- | --- | --- | --- | --- | --- | --- |
|  | **BOOST programme**  **N=248** | | **Best Practice Advice (BPA)**  **N=119** | | |  |
|  | **Mean (SE)** | **N (%)** | **Mean (SE)** | **N (%)** |  | |
| **Participant accommodation (no. of nights)** |  |  |  |  |  | |
| Sheltered housing/warden control | 0 (-) | 0 (-) | 0 (-) | 0 (-) | - | |
| Extra care housing | 0 (-) | 0 (-) | 0 (-) | 0 (-) | - | |
| Care home with nursing care | 0 (-) | 0 (-) | 0 (-) | 0 (-) | - | |
| Care home with personal care | 0 (-) | 0 (-) | 0 (-) | 0 (-) | - | |
| Dual Registered home (providing both personal and nursing care) | 0 (-) | 0 (-) | 0 (-) | 0 (-) | - | |
| Rehabilitation ward | 0 (-) | 0 (-) | 0 (-) | 0 (-) | - | |
| General medical ward | 0 (-) | 0 (-) | 0 (-) | 0 (-) | - | |
| Other | 0 (-) | 0 (-) | 0 (-) | 0 (-) | - | |
|  |  |  |  |  |  | |
| **Hospital inpatient services** |  |  |  |  |  | |
| Inpatient days (days) | 0.29 (0.07) | 26 (10.48%) | 0.44 (0.17) | 11 (9.24%) | 0.3339 | |
| Day case (attended) | - | 44 (17.81%) | - | 20 (16.81%) | 0.0564 | |
|  |  |  |  |  |  | |
| **Surgery, n (%)** | **-** | 2 (0.01%) | **-** | 1 (0.01%) | 0.4360 | |
|  |  |  |  |  |  | |
| **Hospital Outpatient Services (no. of visits)** |  | 136 (55.28%) |  | 69 (58.97%) | 0.5080 | |
| Orthopaedic/Spinal Clinic | 0.19 (0.03) | 34 (13.71%) | 0.29 (0.10) | 14 (11.76%) | 0.2910 | |
| Physiotherapy department | 0.46 (0.13) | 18 (7.26%) | 0.29 (0.09) | 13 (10.92%) | 0.4134 | |
| Accident and Emergency | 0.09 (0.03) | 17 (6.85%) | 0.10 (0.03) | 10 (8.40%) | 0.8499 | |
| Geriatrician | 0.00 (0.00) | 1 (0.40%) | 0.01 (0.01) | 1 (0.84%) | 0.5956 | |
| Other |  | 93 (37.50%) |  | 49 (41.18%) | 0.4980 | |
|  |  |  |  |  |  | |
| **General community-based health services (no. of visits)** |  | 204 (82.93%) |  | 106 (90.60%) | 0.0530 | |
| General practitioner –Office visit | 1.90 (0.14) | 180 (72.58%) | 2.30 (0.22) | 98 (82.35%) | 0.1138 | |
| General practitioner –Home visit | 0.014 (0.09) | 3 (1.21%) | 0.08 (0.08) | 1 (0.84%) | 0.6945 | |
| Other practitioner –Office visit | 0.02 (0.02) | 3 (1.21%) | 0.02 (0.01) | 2 (1.68%) | 0.7764 | |
| Other practitioner –Home visit | - | - | - | - | - | |
| Practice nurse –Office visit | 0.71 (0.09) | 96 (38.71%) | 1.03 (0.19) | 50 (42.02%) | 0.0781 | |
| Practice nurse –Home visit | 0.01 (0.01) | 1 (0.40%) | 0.01 (0.01) | 1 (0.84%) | 0.9793 | |
| District nurse –Office visit | 0.01 (0.01) | 1 (0.40%) | 0.06 (0.05) | 2 (1.68%) | 0.2364 | |
| District nurse –Home visit | 0.04 (0.03) | 2 (0.81%) | 0 (-) | 0 (-) | 0.3293 | |
| Health visitor– Office Visit | - | - | - | - | - | |
| Health visitor– Home Visit | - | - | - | - | - | |
| Incontinence nurse – Office visit | - | - | - | - | - | |
| Incontinence nurse – Home Visit | - | - | - | - | - | |
| Occupational therapist – Office visit | - | - | - | - | - | |
| Occupational therapist – Home Visit | 0.01 (0.01) | 2 (0.81%) | 0.02 (0.01) | 2 (1.68%) | 0.7594 | |
| Physiotherapist – Office visit | 0.48 (0.16) | 16 (6.45%) | 0.25 (0.08) | 12 (10.08%) | 0.3167 | |
| Physiotherapist – Home Visit | - | - | - | - | - | |
| Alternative medicine/therapist – Office visit | 0.05 (0.03) | 5 (2.02%) | 0.13 (0.07) | 4 (3.36%) | 0.2200 | |
| Alternative medicine/therapist – Home visit | 0.01 (0.01) | 1 (0.40%) | 0 (-) | 0 (-) | 0.4892 | |
| Chiropodist – Office visit | 0.13 (0.04) | 17 (6.85%) | 0.13 (0,06) | 8 (6.72%) | 0.9840 | |
| Chiropodist – Home visit | 0.04 (0.03) | 4 (1.61%) | 0.14 (0.06) | 5 (4.20%) | 0.0649 | |
| Other – no of visits | 0.23 (0.11) | 17 (6.85%) | 0.19 (0.07) | 9 (7.56%) | 0.8274 | |
| **Social care services (no. of visits)** |  | 2 (0.81%) |  | 6 (5.04%) | 0.009 | |
| Care manager – Office Visit | - | - | - | - | - | |
| Care manager – Home Visit | - | - | - | - | - | |
| Social worker – Office Visit | - | - | - | - | - | |
| Social worker – Home Visit | 0.02 (0.02) | 1 (0.40%) | 0 (-) | 0 (-) | 0.4892 | |
| Home care worker – Office Visit | - | - | - | - | - | |
| Home care worker – Home Visit | 1.45 (1.45) | 1 (0.40%) | 0.35 (0.35) | 1 (0.84%) | 0.6031 | |
| Carer support worker – Office Visit | 0 (-) | 0 (-) | 0.01 (0.01) | 1 (0.84%) | 0.1491 | |
| Carer support worker – Home Visit | 0 (-) | 0 (-) | 0.06 (0.04) | 3 (2.52%) | 0.0200 | |
| Sitting scheme – Home Visit | - | - | - | - | - | |
| Meals on wheels (no. received) | - | - | - | - | - | |
| Laundry service (no. of times service used) | - | - | - | - | - | |
| Self-help group for participant – Office Visit | 0 (-) | 0 (-) | 0.08 (0.07) | 2 (1.68%) | 0.1070 | |
| Self-help group for participant – Home Visit | - | - | - | - | - | |
| Self-help group for carer – Office Visit | - | - | - | - | - | |
| Self-help group for carer – Home Visit | - | - | - | - | - | |
|  |  |  |  |  |  | |
| **Equipment, adaptations and repairs, n (%)** |  | 16 (6.45%) |  | 22 (18.49%) | <0.001 | |
|  |  |  |  |  |  | |
| **Medications, n (%)** |  |  |  |  | 0.1130 | |
| Number of participants with: |  |  |  |  |  | |
| One medication |  | 15 (6.05%) |  | 10 (8.40%) |  | |
| Two medications |  | 33 (13.31%) |  | 8 (6.72%) |  | |
| Three medications |  | 22 (8.87%) |  | 16 (13.45%) |  | |
| More than three medications |  | 123 (49.60%) |  | 66 (55.46%) |  | |
|  |  |  |  |  |  | |
| **Time off work (days)** |  |  |  |  |  | |
| Participant | 0.14 (0.10) | 2 (0.81%) | 0.08 (0.07) | 2 (1.68%) | 0.6811 | |

**Table S6 Health resource use by trial allocation, category and study period for complete cases at 12months post-randomisation**

| **Resource Category (unit)** | **Treatment arm** | | | | | **P value** |
| --- | --- | --- | --- | --- | --- | --- |
|  | **BOOST programme**  **N=227** | | **Best Practice Advice (BPA)**  **N=114** | | |  |
|  | **Mean (SE)** | **N (%)** | **Mean (SE)** | **N (%)** |  | |
| **Participant accommodation (no. of nights)** |  |  |  |  |  | |
| Sheltered housing/warden control | 0 (-) | 0 (-) | 0 (-) | 0 (-) | - | |
| Extra care housing | 0 (-) | 0 (-) | 0 (-) | 0 (-) | - | |
| Care home with nursing care | 0 (-) | 0 (-) | 0 (-) | 0 (-) | - | |
| Care home with personal care | 0 (-) | 0 (-) | 0 (-) | 0 (-) | - | |
| Dual Registered home (providing both personal and nursing care) | 0 (-) | 0 (-) | 0 (-) | 0 (-) | - | |
| Rehabilitation ward | 0 (-) | 0 (-) | 0 (-) | 0 (-) | - | |
| General medical ward |  | 1 (0.44%) |  | 0 (-) | 0.4780 | |
| Other |  | 0 (-) |  | 3 (2.63%) | 0.014 | |
|  |  |  |  |  |  | |
| **Hospital inpatient services** |  |  |  |  |  | |
| Inpatient days (days) | 0.80 (0.24) | 35 (15.42%) | 0.89 (0.36) | 18 (15.79%) | 0.8360 | |
| Day case (attended) | - | 50 (22.03%) | - | 19 (16.67%) | 0.2450 | |
|  |  |  |  |  |  | |
| **Surgery, n (%)** | **-** | 8 (3.52%) | **-** | 1 (0.88%) | 0.1500 | |
|  |  |  |  |  |  | |
| **Hospital Outpatient Services (no. of visits)** |  | 133 (59.38%) |  | 73 (64.60%) | 0.3530 | |
| Orthopaedic/Spinal Clinic | 0.39 (0.08) | 46 (20.26%) | 0.38 (0.09) | 23 (20.18%) | 0.9358 | |
| Physiotherapy department | 0.26 (0.07) | 20 (8.81%) | 0.24 (0.12) | 7 (6.14%) | 0.8632 | |
| Accident and Emergency | 0.11 (0.03) | 20 (8.81%) | 0.10 (0.04) | 9 (7.89%) | 0.7576 | |
| Geriatrician | 0.01 (0.01) | 2 (0.88%) | 0 (-) | 0 (-) | 0.3162 | |
| Other |  | 91 (41.09%) |  | 58 (50.88%) | 0.0580 | |
|  |  |  |  |  |  | |
| **General community-based health services (no. of visits)** |  | 201 (88.55%) |  | 103 (91.15%) | 0.4620 | |
| General practitioner –Office visit | 2.18 (0.16) | 177 (77.97%) | 2.54 (0.35) | 91 (79.82%) | 0.2712 | |
| General practitioner –Home visit | 0 (-) | 0 (-) | 0.09 (0.09) | 1 (0.88%) | 0.1585 | |
| Other practitioner –Office visit | 0 (-) | 0 (-) | 0.09 (0.09) | 1 (0.88%) | 0.1585 | |
| Other practitioner –Home visit | - | - | - | - | - | |
| Practice nurse –Office visit | 0.90 (0.12) | 93 (40.97%) | 1.30 (0.29) | 53 (46.49%) | 0.1287 | |
| Practice nurse –Home visit | - | - | - | - | - | |
| District nurse –Office visit | 0 (-) | 1 (0.44%) | 0.05 (0.05) | 1 (0.88%) | 0.1585 | |
| District nurse –Home visit | 0.08 (0.05) | 4 (1.76%) | 0.04 (0.03) | 2 (1.75%) | 0.5471 | |
| Health visitor– Office Visit | 0.00 (0.00) | 1 (0.44%) | 0.01 (0.01) | 1 (0.88%) | 0.6196 | |
| Health visitor– Home Visit | - | - | - | - | - | |
| Incontinence nurse – Office visit | 0.01 (0.01) | 1 (0.44%) | 0 (-) | 0 (-) | 0.4793 | |
| Incontinence nurse – Home Visit | - | - | - | - | - | |
| Occupational therapist – Office visit | - | - | - | - | - | |
| Occupational therapist – Home Visit | 0.02 (0.01) | 2 (0.88%) | 0 (-) | 0 (-) | 0.3162 | |
| Physiotherapist – Office visit | 0.34 (0.13) | 15 (6.61%) | 0.02 (0.01) | 2 (1.75%) | 0.0904 | |
| Physiotherapist – Home Visit | 0.04 (0.04) | 2 (0.88%) | 0 (-) | 0 (-) | 0.4295 | |
| Alternative medicine/therapist – Office visit | 0.18 (0.11) | 4 (1.76%) | 0.06 (0.05) | 3 (2.63%) | 0.4540 | |
| Alternative medicine/therapist – Home visit | - | - | - | - | - | |
| Chiropodist – Office visit | 0.19 (0.06) | 15 (6.61%) | 0.13 (0.05) | 8 (7.02%) | 0.5400 | |
| Chiropodist – Home visit | 0.07 (0.03) | 5 (2.20%) | 0.09 (0.05) | 3 (2.63%) | 0.7249 | |
| Other – no of visits | 0.28 (0.09) | 21 (9.25%) | 0.49 (0.21) | 13 (11.40%) | 0.2835 | |
| **Social care services (no. of visits)** |  |  |  |  |  | |
| Care manager – Office Visit | 0 (-) | 0 (-) | 0.01 (0.01) | 1 (0.88%) | 0.1585 | |
| Care manager – Home Visit | - | - | - | - | - | |
| Social worker – Office Visit | 0.00 (0.00) | 1 (0.44%) | 0 (-) | 0 (-) | 0.4793 | |
| Social worker – Home Visit | 0.01 (0.01) | 1 (0.44%) | 0 (-) | 0 (-) | 0.4793 | |
| Home care worker – Office Visit | - | - | - | - | - | |
| Home care worker – Home Visit | - | - | - | - | - | |
| Carer support worker – Office Visit | - | - | - | - | - | |
| Carer support worker – Home Visit | 0 (-) | 0 (-) | 0.03 (0.03) | 1 (0.88%) | 0.1585 | |
| Sitting scheme – Home Visit | - | - | - | - | - | |
| Meals on wheels (no. received) | - | - | - | - | - | |
| Laundry service (no. of times service used) | - | - | - | - | - | |
| Self-help group for participant – Office Visit | - | - | - | - | - | |
| Self-help group for participant – Home Visit | - | - | - | - | - | |
| Self-help group for carer – Office Visit | - | - | - | - | - | |
| Self-help group for carer – Home Visit | - | - | - | - | - | |
|  |  |  |  |  |  | |
| **Equipment, adaptations and repairs, n (%)** |  | 23 (10.13%) |  | 9 (7.89%) | 0.4040 | |
|  |  |  |  |  |  | |
| **Medications, n (%)** |  |  |  |  | 0.2730 | |
| Number of participants with: |  |  |  |  |  | |
| One medication |  | 26 (11.45%) |  | 11 (9.65%) |  | |
| Two medications |  | 26 (11.45%) |  | 13 (11.40%) |  | |
| Three medications |  | 22 (9.69%) |  | 8 (7.02%) |  | |
| More than three medications |  | 136 (59.91%) |  | 65 (57.02%) |  | |
|  |  |  |  |  |  | |
| **Time off work (days)** |  |  |  |  |  | |
| Participant | 0.40 (0.40) | 1 (0.44%) | 1.58 (1.58) | 1 (0.88%) | 0.3447 | |

**Table S7 Economic costs by trial allocation arm and cost component category for the entire follow-up period for the NHS PSS perspective among complete cases (£, 2018-19 prices)**

| **Cost category** | **Treatment arm, mean cost (£) (SE)** |  | **Mean cost (£) difference** | ***p*-value ^i^** | **Bootstrap 95% CI ^ii^** |
| --- | --- | --- | --- | --- | --- |
|  | **BOOST programme** | **Best Practice Advice (BPA)** |  |  |  |
|  | (n=150) | (n=70) |  |  |  |
| NHS and PSS |  |  |  |  |  |
| Intervention Costs | 395.52 (9.95) | 73.91 (2.83) | 321.61 | <0.0001 | (301.25 to 341.98) |
| Hospital inpatient services | 319.22 (120.43) | 407.17  (163.15) | -87.95 | 0.665 | (-487.83 to 311.93) |
| Hospital day care services | 92.98 (27.42) | 36.70 (17.27) | 56.28 | 0.084 | (-7.58 to 120.13) |
| Hospital outpatient services | 360.54 (109.06) | 391.71  (110.78) | -31.16 | 0.841 | (-333.43 to 271.10) |
| General community-based health services |  | 161.95  (14.16) | 5.00 | 0.818 | (-38.45 to 48.45) |
| Community-based social care services | 0.30 (0.30) | 4.62 (2.69) | -4.32 | 0.115 | (-9.62 to 0.97) |
| Equipment, adaptations/repairs | 1.49 (0.71) | 1.82(1.03) | -0.33 | 0.792 | (-2.80 to 2.14) |
| Concomitant/prescription medications | 305.27 (27.51) | 381.71 (64.09) | -76.45 | 0.276 | (-212.08 to 59.19) |
| **Total (NHS and PSS) (excluding intervention costs)** | **1246.76 (176.02)** | **1385.69 (208.84)** | **-138.93** | **0.612** | **(-678.71 to 400.85)** |
| **Total (NHS and PSS) (including intervention costs)** | **1642.28 (176.90)** | **1459.60**  **(209.36)** | **182.68** | **0.506** | **(-344.03 to 709.40)** |

^i^ *p*-value calculated using the student’s t-test, two-tail unequal variance

^ii^Non-parametric bootstrap estimation using 10,000 replications, bias corrected used

**Table S8 Economic costs by trial allocation arm and cost component category for the entire follow-up period for the societal perspective among complete cases (£, 2018–19 prices)**

| **Cost category** | **Treatment arm, mean cost (£) (SE)** | | **Mean cost (£) difference** | ***p*-value ^i^** | **Bootstrap 95% CI ^ii^** |
| --- | --- | --- | --- | --- | --- |
|  | **BOOST programme** | **Best Practice Advice (BPA)** |  |  |  |
|  | (n=150) | (n=70) |  |  |  |
| Total (NHS and PSS) (including intervention costs) | 1642.28 (176.90) | 1459.60  (209.36) | 182.68 | 0.506 | (-344.03 to 709.40) |
| Broader societal |  |  |  |  |  |
| Privately provided health services | 6.97 (3.73) | 6.11 (3.82) | 0.86 | 0.872 | (-12.45 to 25.14) |
| Medications | 5.76 (1.58) | 7.08 (1.33) | -1.32 | 0.524 | (-5.42 to 2.52) |
| Patient equipment | 24.35 (15.56) | 33.23 (14.64) | -8.87 | 0.678 | (-40.16 to 32.16) |
| Patient travel | 13.22 (2.34) | 22.87 (6.58) | -9.66 | 0.170 | (-18.72 to 5.32) |
| Time off work | 5.73 (4.05) | 128.30 | -122.57 | 0.146 | (-188.51 to 53.05) |
| Other Societal Costs | 104.91 (56.44) | 184.55 | -79.64 | 0.553 | (-276.58 to 83.81) |
| **Total Broader Societal** | **160.95 (59.90)** | **382.14** | **-221.19** | **0.243** | **(-591.72 to 149.34)** |
| **Total Societal** | **1712.57 (236.28)** | **1512.56 (260.69)** | **200.01** | **0.571** | **(-566.94 to 849.08)** |

^i^ *p*-value calculated using the student’s t-test, two-tail unequal variance

^ii^Non-parametric bootstrap estimation using 10,000 replications, bias corrected used

**Table S9 Patient reported EQ-5D-5L utility scores and QALYs (Imputed Analysis)**

| **Variable** | **Treatment arm** | **N** | **Mean (SE)** | **Mean Difference**  **(95% CI)** |
| --- | --- | --- | --- | --- |
|  |  |  |  |  |
| EQ-5D-5L utility scores at 6 months post-randomisation | Group Physiotherapy | 292 | 0.598 (0.013) | 0.039 (0.008 to 0.071) |
|  | Best Practice Advice | 143 | 0.637 (0.009) |  |
|  |  |  |  |  |
| EQ-5D-5L utility scores at 12 months post-randomisation | Group Physiotherapy | 292 | 0.607 (0.015) | 0.009 (-0.027 to 0.045) |
|  | Best Practice Advice | 143 | 0.616 (0.010) |  |
|  |  |  |  |  |
| **QALYs (EQ-5D-5L)** | **Group Physiotherapy** | **292** | **0.620 (0.009)** | **0.021 (0 to 0.044)** |
|  | **Best Practice Advice** | **143** | **0.599 (0.006)** |  |
|  |  |  |  |  |


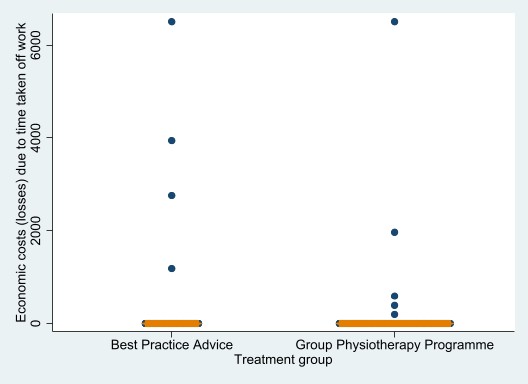


Figure S1 Economic losses due to lost days of work by participants and/ carers (£, 2018-19)

**References**

1. NHS Improvement. Reference Costs 2016-2017. In: Department of Health, editor. London, 2018.

2. Curtis LA, Burns, A. Unit Costs of Health and Social Care 2019. University of Kent, Canterbury: Personal Social Services Research Unit; 2019.

3. Curtis LA, Burns, A. Unit Costs of Health and Social Care 2015. University of Kent, Canterbury: Personal Social Services Research Unit; 2015.

4. Curtis LA. Unit costs of health and social care University of Kent, Canterbury: Personal Social Services Research Unit,; 2010.

5. Curtis LA. Unit costs of health and social care University of Kent, Canterbury: Personal Social Services Research Unit; 2014.

6. NHS. NHS Supply Chain. London: NHS Digital; 2018.

1. Costs were inflated or deflated to 2018/19 values using the currently recommended NHSCII index [↑](#endnote-ref-1)
2. Estimates derived by cross-referencing participant’s/carer’s profession as captured in questionnaire to earnings data reported in the annual survey of hours and earnings (ASHE) [↑](#endnote-ref-2)
3. Inclusive for each site: costs of ankle weights, dumb bells and stand, weighted vests, stopwatch, lap counter, egg timer, plastic storage crates, large traffic cones, wheeled trolley. The capital cost of each item was converted into an annual equivalent cost by annuitizing the costs over the expected life of each equipment using a discount rate of 3.5% per annum. [↑](#endnote-ref-3)
4. Costs of individual 60min appointment conducted prior to attending the programme to set individualised exercise and walking set targets for the group sessions [↑](#endnote-ref-4)
5. Group session costs (maximum 12 per group) calculated as indicated in table x above and include administration costs [↑](#endnote-ref-5)
6. Follow-up costs calculated as physiotherapist’s hourly wage rate multiplied by average time (in hours) spent on call with a participant as reported by each site. [↑](#endnote-ref-6)
7. Calculated as total staff cost of delivering the group sessions for a group within a site divided by the number of participants per group multiplied by mean number of sessions attended. Costs included are physiotherapists and assistants’ costs of delivering the group sessions that last 90±15min. [↑](#endnote-ref-7)
8. Calculated as total staff cost of delivering the group sessions for a group within a site divided by the number of participants per group multiplied by mean number of sessions attended. Costs included are physiotherapists and assistants’ costs of delivering the group sessions that last 90±15min. [↑](#endnote-ref-8)
9. Administration costs included: (i) additional time spent on administrative tasks by the main physiotherapist at the end of each group session (outside the 90 min) session; (ii) administration time spent prior to classes starting (a once-off cost per group) and (iii) administration time after the group classes were completed (once-off cost per group). [↑](#endnote-ref-9)
10. Total staff costs for group sessions including administrative time costs [↑](#endnote-ref-10)
